# Serial optical coherence microscopy for label-free volumetric histopathology

**DOI:** 10.1038/s41598-020-63460-3

**Published:** 2020-04-21

**Authors:** Eunjung Min, Sungbea Ban, Junwon Lee, Andrey Vavilin, Songyee Baek, Sunwoo Jung, Yujin Ahn, Kibeom Park, Sungwon Shin, SoHyun Han, Hyungjoon Cho, Whaseon Lee-Kwon, Jeehyun Kim, C. Justin Lee, Woonggyu Jung

**Affiliations:** 1 Max Planck Institute for Biological Cybernetics, 72076 Tübingen, Germany; 2 Department of Biomedical Engineering, Ulsan National Institute of Science and Technology (UNIST), Ulsan, 44919 Republic of Korea; 3 Martinos Center for Biomedical Imaging, Charlestown, MA 02129 United States; 4 School of Electronics Engineering, Kyungpook National University, Daegu, 41566 Republic of Korea; 5 Center for Cognition and Sociality, Institute for Basic Science, Daejeon, 34126 Republic of Korea

**Keywords:** Biotechnology, Imaging

## Abstract

The observation of histopathology using optical microscope is an essential procedure for examination of tissue biopsies or surgically excised specimens in biological and clinical laboratories. However, slide-based microscopic pathology is not suitable for visualizing the large-scale tissue and native 3D organ structure due to its sampling limitation and shallow imaging depth. Here, we demonstrate serial optical coherence microscopy (SOCM) technique that offers label-free, high-throughput, and large-volume imaging of *ex vivo* mouse organs. A 3D histopathology of whole mouse brain and kidney including blood vessel structure is reconstructed by deep tissue optical imaging in serial sectioning techniques. Our results demonstrate that SOCM has unique advantages as it can visualize both native 3D structures and quantitative regional volume without introduction of any contrast agents.

## Introduction

The histological examination of excised tissue via optical microscope has been widely used in biological laboratory and clinic, which requires the cumbersome process such as fixation, embedding, sectioning, and staining of biological specimens^1–3^. Although histologic analysis in pathology is a gold-standard to understand tissue morphology, diagnose diseases, and decide treatment course, it is still very time- and labor-intensive and provides only limited tissue information in thin and narrow field of view. In order to overcome current restriction of histopathology, several optical imaging techniques have been introduced toward rapid and stain-free histopathology of fresh tissues^4–10^. However, current methods lack the ability to visualize diagnostic pathologic feature over large areas or volumetric context.

Over the past decade, advanced 3D microscopic imaging techniques for high resolution and volumetric anatomy have been demonstrated. 3D microscopic imaging techniques have been initially applied to neuroscience and developmental studies. One of representative attempts of fully automated bright field microscopy^11^, single photon microscopy^12^ and two-photon microscopy combined with tissue sectioning method^13^ were successfully used to reconstruct fine neuronal network and anatomical tracings in a whole mouse brain. While these techniques can generate a complete dataset such as projectomes and connectomes, the high cost of equipment, the huge amount of time needed for tissue processing and fluorescent labeling, and massive data collection and signal processing make this technique difficult to be widely available for pathology applications.

Another novel approach for volumetric imaging is based on light-sheet fluorescence microscopy and chemically clearing tissue^14–21^. As the effective clearing agents are available, the deep tissue optical imaging has become feasible through either the refractive index matching of tissue or removal of the highly scattered biological components like lipid. A number of tissue clearing techniques^14,19^ have been introduced and successfully combined with light-sheet microscopy to unveil the cellular connectivity at large areas. This approach allows fast volumetric imaging without the need for mechanical sectioning. However, it requires careful sample preparation for clearing protocols as well as long incubation for immunostaining, which can often distort the physical volume of the entire 3D specimens and thus can restrict its utility for pathology. An ideal 3D optical imaging technique for histopathology would be able to image unlabeled and unstained fresh specimens and provide high throughput quantitative information as well as native 3D context.

Optical coherence microscopy (OCM)^22–26^ could be an alternative imaging modality for high throughput histopathology. OCM is based on a coherent gating technique of optical interferometry, and utilizes the endogenous back-scattering signal. Thus, it does not require chemical labeling, staining or external contrast agents. OCM is also suitable for deep tissue imaging, because OCM uses near infrared light that reaches deeper into biological tissues than visible light can reach. To date, optical imaging based on coherent gating technique has widely been applied in the clinic to diagnose diseases of various tissues^27,28^. In particular, tomographic imaging has an important role as optical biopsy, because it can provide a real-time structural information non-invasively at spatial resolutions similar to that of a standard histological section taken from a biopsy sample. Characteristics of coherent gating technique are inherently well-suited for ideal histopathologic imaging, and it is particularity useful as an alternative to histological tissue observation when it is not practical to take specimens for histological processing or when large areas of tissue are needed to be investigated. Though prior studies presented the feasibility and potential of OCM for the histopathology^29,30^, there is a lack of investigation to demonstrate 3D image and structural analysis of the whole organs. Here, we report the very first serial optical coherence microscopy (SOCM), a combination of coherent gating technique of optical interferometry and serial sectioning technique for high throughput whole mouse organ imaging.

## Results

### SOCM system

**Figure 1:**
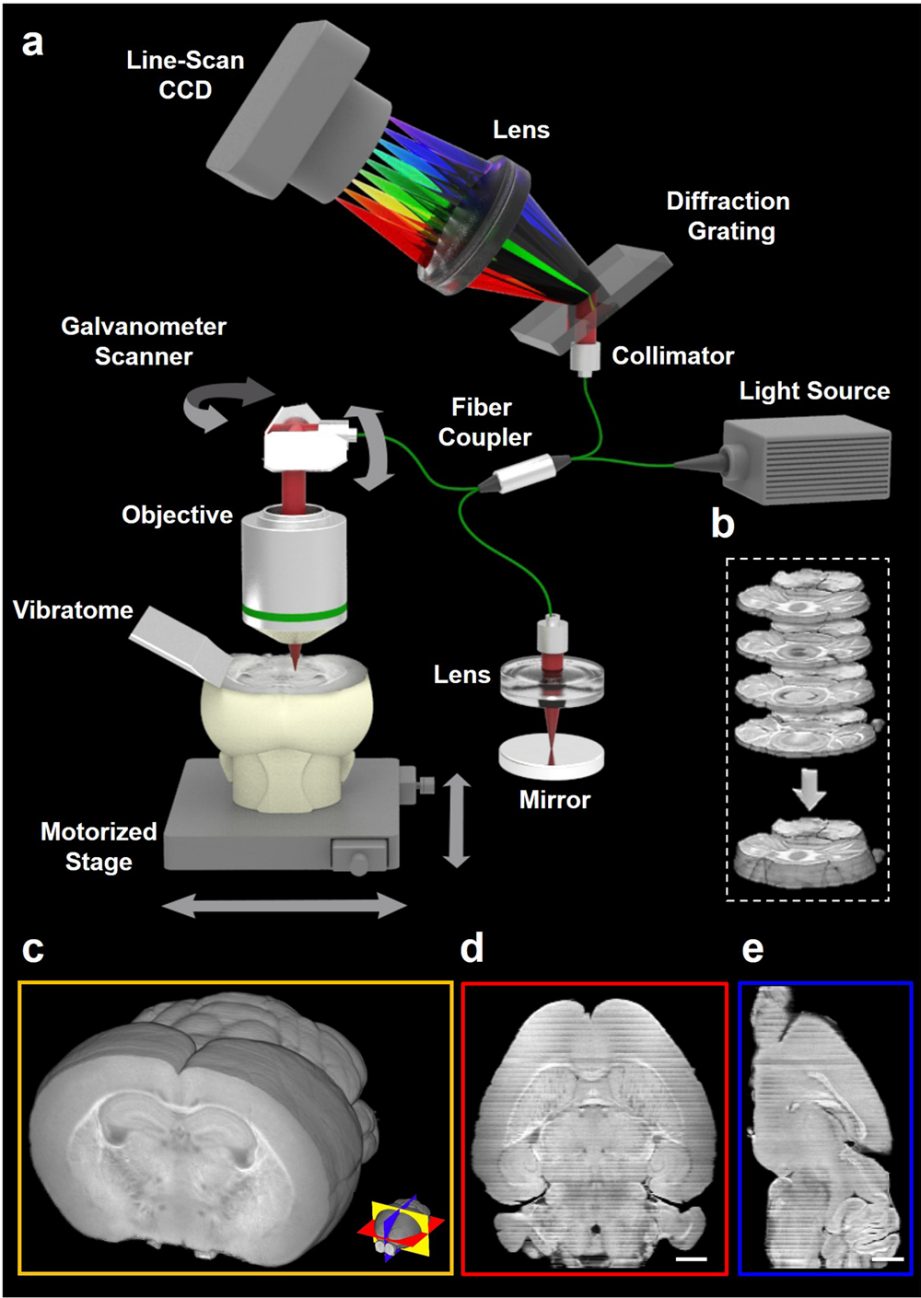
SOCM system for whole organ imaging. (**a**) Schematic of SOCM system based on coherent gating imaging and serial sectioning technique. The process of imaging and sectioning are repeated until whole organ image set is completed. (**b**) Brain slices sequentially sectioned with 200 μm thickness using a vibratome. (**c**) A whole brain image reconstructed with dataset of 60 coronal sections (**d,e**) Cross sectional images of the axial and sagittal plane obtained after whole brain image reconstruction. Scale bars, 1 mm.

The OCM system in our study is based on a spectral domain interferometer with 1300 nm wavelength light source (Fig. 1a). The axial and lateral resolutions were measured as 9.9 μm and 10.7 μm in air. SOCM for large-scale and volumetric imaging follows three essential steps: (i) tissue embedding in agarose gel, (ii) sectioning of specimens with vibratome, and (iii) imaging by automated OCM. The process of imaging and sectioning is repeated until whole organ image set is completed. Since deep tissue imaging capability of SOCM has benefit to reduce the total number of physical sectioning of organ for imaging, we are able to minimize the inclusion of image distortion during image stitching process for large-scale reconstruction.

The imaging capability of SOCM was initially evaluated for the reconstruction of whole mouse brain. For the whole brain imaging, 60 tissue sections of mouse brain in coronal plane at 200 μm thickness were acquired (see Fig. S1 in the Supplementary Materials). The total image acquisition time for an entire brain was less than 1 hour, which is perhaps the fastest time compared to any other reported serial optical imaging techniques. Under our new alignment algorithm, each sectioned image was obtained with 20% overlapping region with the next section along the z-axis (depth), which helps to minimize distortion by tissue slicing and thus optimizes image reconstruction (Fig. 1b). Through this process, 3D image of the whole brain was reconstructed (see Fig. S2 and Video S1 in the Supplementary Materials). After reconstruction, the whole brain can be visualized along any planes including coronal, sagittal, and axial planes (Fig. 1c–e).

### Whole-brain imaging

**Figure 2:**
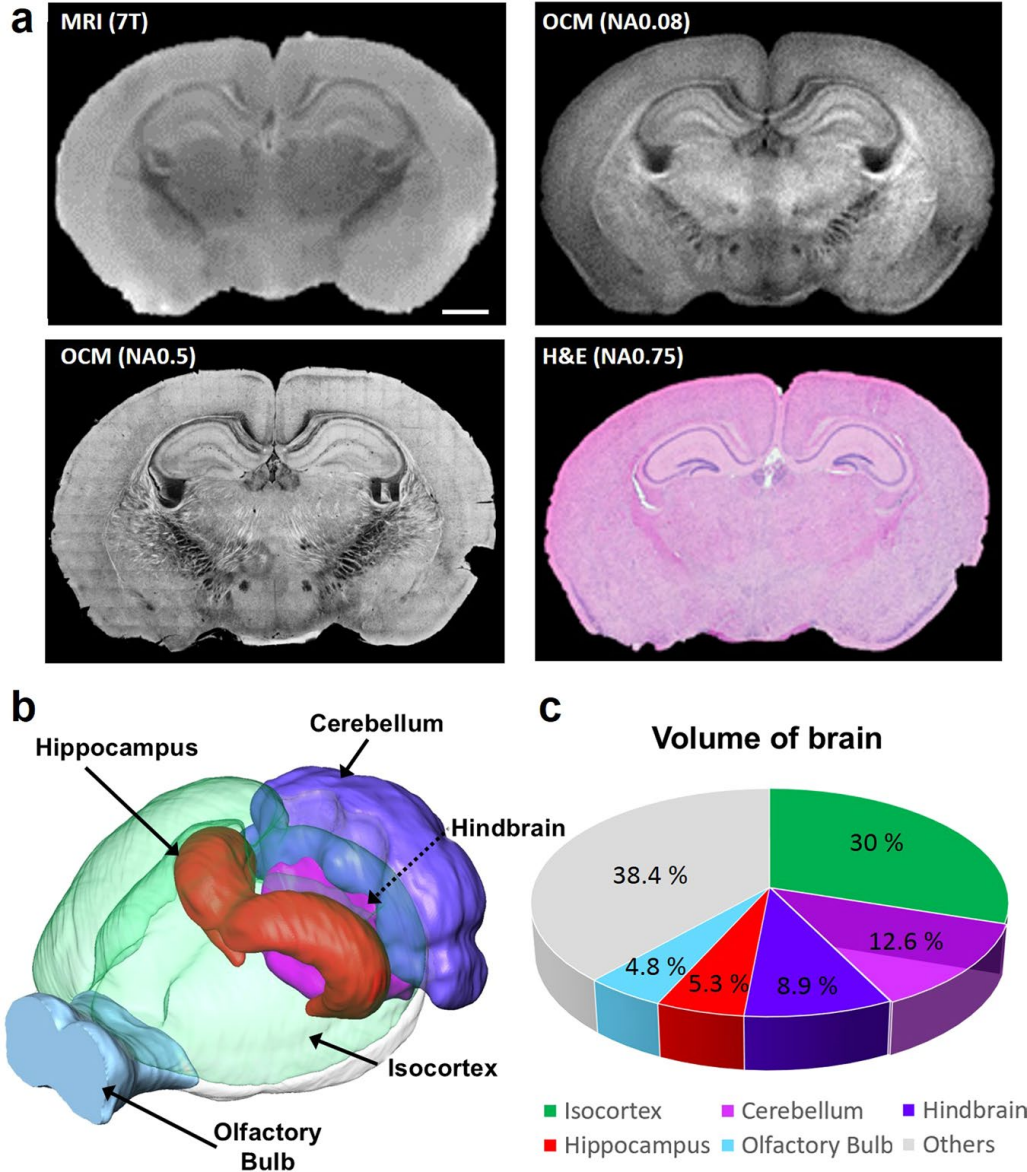
Comparison images of brain tissue and quantitative SOCM image. (**a**) Comparison of MRI and NA 0.08 SOCM image of a mouse brain tissue (up), comparison of H&E histology and NA 0.5 SOCM image of a mouse brain tissue (bottom) (**b**) Visualization of reconstructed whole mouse brain and rendered segmentation of regional volume (**c**) Volume measurement of whole brain with pie chart. Scale bars, 1 mm.

The reconstructed SOCM images of mouse brain in coronal plane clearly delineate structural details such as cortex, hippocampus, and corpus callosum (Fig. 1). SOCM images were directly compared with images of the same brain sections taken by magnetic resonance imaging (MRI) (upper part of the Fig. 2a). Additionally, the high resolution SOCM image was also compared with the histology microscope image as shown at the bottom of Fig. 2a. To obtain high resolution images, we used short wavelength of 840 nm and high numerical aperture objective lenses (NA 0.5). SOCM images of various brain regions showed a significantly higher contrast and acuity compared to those of the other two techniques. (see Figs. S3 and S4 in the Supplementary Materials). By using the high resolution SOCM, we were clearly able to resolve fiber-rich areas due to enhanced contrast arising from higher scattering coefficient in the individual myelinated fiber bundle than that of the surrounding regions^31^. The individual myelinated fibers are more clearly visible as the numerical aperture increases (see Fig. S5 in the Supplementary Materials). Stereological analysis of histological sections has been used for several decades for measuring the hippocampal volume of mice. Even though stereology provides practical approach for extracting quantitative and volumetric information of tissue, it offers crudely estimated volume fraction. On the other hand, using SOCM, we are able to segment whole brain based on Allen institute 3D atlas map and divide it into five different major regions of the brain (Fig. 2b,c and Video S2 in the Supplementary Materials). Our volumetric analysis provides regional volumes of brain with sufficient resolution and contrast which has been restricted by stereological analysis requiring significant amount of labor and time.

### Whole-kidney imaging

**Figure 3:**
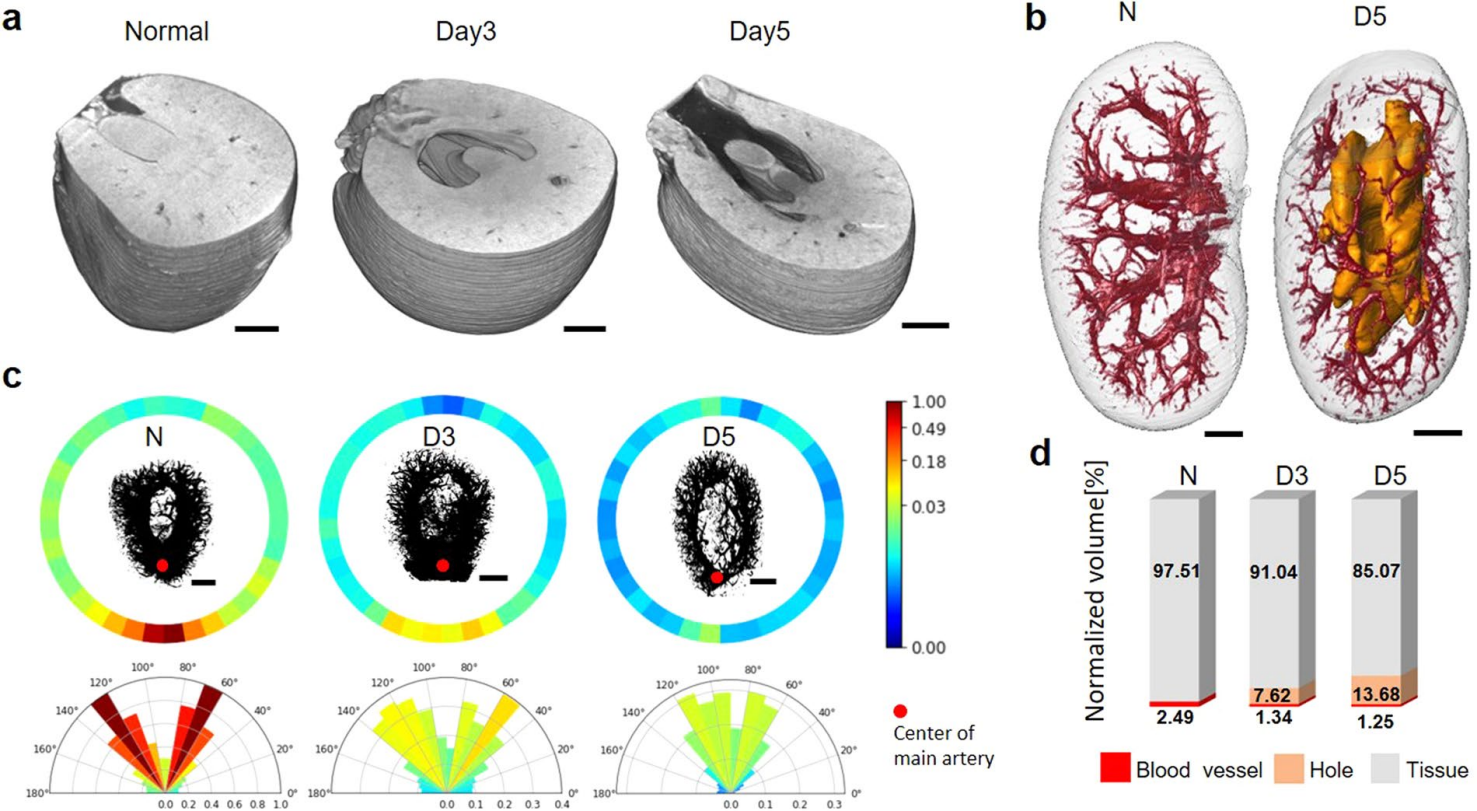
Whole mount kidney images using SOCM. (**a**) Volumetric visualization of kidney structure at transvers plane as the stage of UUO develops (**b**) Visualization of whole kidney structure including vessel architecture in wild type and 5 day of UUO. Blood vessel networks and empty hole was denoted by red and orange color, respectively. The outermost transparent shell with its entire morphological shape is presented with empty hole colored by yellow. (**c**) Orthogonal projection analysis for extracting quantitative vessel distribution. Scale bars, 1 mm (**d**) Quantitative volume analysis of major segment such as renal tissue, blood vessel and empty hole.

SOCM was used to identify the pathology for one of typical renal disease, unilateral ureteral obstruction (UUO)^32^. In UUO, constricted urinary tract induces the slow flow of urine out of kidney as stage develops, with blocked toxic urine resulting in irreversible damage and permanent functional failure^33^. Thus, it is essential to monitor the gradual and morphological change of inner kidney in UUO studies. However, desired volumetric structure is hard to be addressed by the histological examination alone due to the nature of thin slice imaging. Conventional imaging modalities such as CT and MRI also has limitation to visualize the whole inner structure of kidney, not only due to its resolution, but also due to the blockage of ureter and constriction of vessel network accessing contrast agent^34,35^. In this work, native 3D morphological feature of kidney such as renal cortex, outer medulla, inner medulla, pelvis and vessel was successfully delineated by SOCM as shown in Fig. 3a,b (see Fig. S6 and Video S3 in the Supplementary Materials). Whole kidneys at different stages of UUO were transversely sectioned in the same manners as completed in brain reconstruction (see Fig. S7 in the Supplementary Materials). The total image acquisition time for an entire kidney was less than 1 hour. In order to visualize and quantify the volumetric blood vessel context, we utilized a customized vessel tracking software (see Fig. S8a–d in the Supplementary Materials). The change in blood vessel volume was analyzed by projection methods as shown in the upper part of the Fig. 3c. From the 3D vessel image, we obtained the 2D projection map by adding up all the intensity values of the images in a direction perpendicular to the coronal plane. The intensity values were then summed again every 10 degrees for the center of the projection image, normalized and displayed in a round band (see Fig. S8e,f in the Supplementary Materials). The value closed to 1 means that blood vessels are concentrated in that area. On the inner surface of the band, the density of blood vessels is shown by max projection image. Furthermore, from the 2D sum projection map obtained above, the intensity value is added every 10 degrees around the center of main artery which is indicated with red dot in Fig. 3c, and plotted in the half polar as shown at the bottom of Fig. 3c. In Fig. 3d, percentage shows that blood vessels shrink as the hole gets bigger. These results statistically demonstrate that the renal dysfunction of UUO due to the blockage of urinary tract shows as the low vessel density at pelvis and medulla. UUO of kidney commonly results in interstitial fibrosis and morphological alteration including vessel deformation^36^. The SOCM identifies the increased volume of empty hole as well as the decreased volume of blood vessel network (Fig. S9 in the Supplementary Materials).

## Discussion

In the present study we have developed SOCM, a new imaging platform for volumetric histopathology. Our results demonstrate that SOCM has unique advantages over the current histopathological techniques with its ability to visualize native 3D structures and to provide quantitative regional volume without any labeling or contrast agents. In the current feasibility study, we evaluated the potential of SOCM in mouse brain and kidney, but it could be well adapted to other organs such as liver, spinal cord, heart and skin. In principle, 3D histopathology by SOCM can be extended to even larger animals including humans. Moreover, we can extract statistically meaningful results from imaging multiple orangs in a short time. However, imaging acquisition time and resolution would be enhanced more when SOCM is integrated with FFOCM^29^ and micro OCM^30^. Future technical improvements and potential applications of SOCM promise exciting new possibilities especially when user-friendly automated SOCM can be combined with deep learning based image processing. Taken together, fully engineered SOCM would allow investigation of volumetric and anatomical alterations associated with various diseases, and provide paradigm shift toward digital histopathology.

## Method

### Brain Tissue Specimens

Animal procedures described here were approved by the Ulsan National Institute of Science and Technology (UNISTIACUC-17-10) and carried out in accordance with Institutional Animal Care and Use Committee standards. In this study, the brain of four weeks C57BL/6 mice was used. The mice were deeply anesthetized by injection of the zoletil (30 mg/kg) and rompun (10 mg/kg). It was transcardially perfused with 30 ml PBS and followed by 60 ml 4% paraformaldehyde in PBS. For fixation, brains were dissected out and incubated in the 4% paraformaldehyde at 4 °C for 24 hours. The brains were then washed in PBS and embedded in 2–3% melted agarose gel using cube shaped mold.

### UUO model and renal tissue preparation

In order to induce unilateral ureteral obstruction (UUO) in 8-weeks-old male C57BL/6 mice, right kidney was exposed for the right flank incision site after the mice were anesthetized with same condition in brain study. The ureter tract at the closest position to the renal pelvis was completely ligated using a black 6–0 silk tie. Sham-operated mice underwent the same surgical procedure except for the ureter ligation. Mice were then sacrificed at various intervals allowing the study of altered renal morphology. Kidneys were harvested on 3 days and 5 days after UUO was surgically induced by ureteral blockage. Vascular perfusion was performed during each kidney harvest, and the tissues were stored in fixation solution of 10% neutral buffered formalin for the SOCM imaging.

### SOCM

Serial optical coherence microscopy (SOCM) is a combined technique between optical coherence microscopy (OCM) and serial block-face imaging technique. OCM setup was constructed based on Michelson interferometry, particularly operated in spectral domain. It was composed of four parts: source, sample, reference and detection parts. Broadband optical light source having center wavelength of 1300 nm with spectral bandwidth 70 nm (Exalos, EXS210046-02) which offers 9.9 μm the depth resolution in air. The source beam was divided by beam splitter into sample and reference arms. Returning two partially coherent light beams meet and interfere each other. The interference signal that contains depth information was detected by the spectrometer composed of volume phase holographic grating (Wasatch Photonics, 1200 lpm @1300 nm), focusing lens and CCD (Sensors Unlimited, SU-LDH2).

In the sample arm, a fixed and agar-embedded mouse organ was placed in a custom designed mount. The mount was made up of container that is filled with water to keep the bio-specimen in moisture during imaging, and reduce back-reflection from the surface of the organ. Once the mouse organ was properly mounted, 2D scanning was performed with xy-galvano scanner (Thorlabs, GVS102) and cross-sectional image of the tissue was obtained using NA 0.08 lens. The lateral resolution in air was 10.7 μm, and confocal parameter was 0.14 mm. Custom algorithm built with LABVIEW was used for imaging and collecting the data. The field of view of cross-sectional image was 13 mm × 1.8 mm (2000×512 pixels). The coronal section images were then obtained after 3D reconstruction, and the field of view of each image was 13 mm × 10 mm (2600 × 2000 pixels). The mount was designed easily removable from the sample arm and moved to vibratome (Pelco 102) for sectioning without disturbing the bulk tissue. The knife of vibratome cut the upper part of organ after imaging. Imaging and sectioning were repeated until the 3D dataset for whole organ was obtained. The thickness of the section was optimally determined as 200 μm. The overlapped region between adjacent images was 20%. This overlapping region minimizes data loss in the final result. The slice thickness was determined to be close to the depth of field, which enhances image sensitivity and correlation between adjacent images during 3D reconstruction. As a result, a whole brain was reconstructed with only 60 organ slices. Total image acquisition time took less than 1 hour.

### High resolution SOCM

Broadband optical light source having center wavelength of 840 nm with spectral bandwidth 60 nm (Superlum, D-840-HP) and objective with higher numerical aperture (Olympus, UPLFLN 20x/NA 0.5) were used to enhance spatial resolution. The axial and lateral resolutions were calculated as 4 μm and 1 μm, respectively. In addition, xy linear motor stage (Newport, XMS100) was utilized to capture the entire sliced tissue. Size of the single image was 500×500 μm^2^ (500×500 pixels), and 10% at the outskirts of the image were overlapped with adjacent images. Then all the images were stitched side by side using in-house developed mosaic algorithm built with MATLAB. With high resolution SOCM, entire coronal, axial and sagittal sections of a mouse brain were obtained (Figs. S3 and S4 of the Supplementary Materials). The number of tiles for entire coronal, axial and sagittal image were 442, 720 and 435, corresponding image acquisition time was about 3 hrs., 5 hrs. and 3 hrs., respectively. Under this circumstance, high resolution image of tissue was obtained. Additionally, bundle of myelin fibers in a brain tissue was imaged with high NA microscope objectives (Olympus, 10x/NA 0.4, 20x/NA 0.5, 40xW/NA 0.8) as represented in Fig. S5 of the Supplementary Materials. The lateral resolutions for NA 0.4, NA 0.5, NA 0.8 were calculated as 1.3 μm, 1 μm and 0.6 μm, respectively. The total image acquisition time corresponding to NA 0.4, NA 0.5 and NA 0.8 were about 0.6 hrs., 2.2 hrs. and 9.7 hrs., respectively.

### Comparison study with MRI and histology

The brain tissue was imaged using MRI and histological section stained with Hematoxylin and Eosin (H&E), and Nissl and Luxol fast blue (N&L) for correlation study. Same mouse brain was used to make this comparison fair. Prior to OCM imaging, MRI imaging (Bruker Biospec 7 T/16 cm) of coronal, axial and sagittal planes was performed with intact brain *ex vivo*. Before applying magnetic field on the sample, air bubbles inside of the agarose-embedded brain, was degassed, that could cause susceptibility artifact in the MR image. The mouse brain was then carefully mounted in MRI system. The imaging parameters of RARE sequence for T2-weighted imaging were set as follows: TR = 8 s, effective TE = 36 ms, RARE factor = 8, NA (number of average) = 140, NS (number of slice) = 25, FOV = 18×15 × 5 mm^3^, matrix: 360 × 300 × 25, resolution = 50×50×200 μm^3^, and total scan time was 11 hours. To make histological section, standard histology procedure, was followed, including fixation, dehydration, paraffin infiltration, embedding, and sectioning. H&E stain was used to identify nucleus and cytoplasm. N&L stain was used to stain Nissl body and myelin. The stained slices were then imaged using virtual microscope (Olympus, dotSlide microscope 20x/0.75).

### Image processing for high resolution and wide field visualization

In order to generate high-resolution image, mosaic stitching algorithm was developed and implemented with MATLAB. Image acquisition provides a set of M × N image patches in coronal plane. The resolution of single patch was 500 × 500 pixels. Imaging system was designed to make 50 pixels overlap between neighboring patches. However, small displacement was possible due to mechanical reasons. Once the scanning process was finished, obtained patches had to be properly aligned and smoothly merged into a single mosaic image. These tasks could be challenging due to the high noise level, non-uniform intensity distribution caused by lens aberration, large number of images to align and lack of structures in every single patch. Intensity difference of adjacent images may cause abrupt transitions between images. To correct this problem, our algorithm consists of three main steps. First, 20% of input patches with high mean intensity and low intensity variance were analyzed to create global intensity correction image. Then we subtracted this image from all patches which help to reduce effect of non-uniform intensity distribution inside the patch image. After initial intensity correction, patches were aligned based on normalized cross-correlation (NCC) of overlapped parts. Finally, sharp transitions between neighboring patches were localized and smoothed. In addition, resulted mosaic image was post-processed in order to increase its contrast.

### Image processing for whole mount 3D reconstruction

In order to visualize the whole organ structure, unique 3D reconstruction protocol for SOCM was developed as shown in Fig. S2 of the Supplementary Materials. The first step of 3D data processing starts from local noise reduction, based on combination of median and average filters, applied to each cross-sectional image independently. Since volumetric data were inherently discarded over the axial direction due to the out-of-focus and high scattering, the attenuation correction was considered to reduce the effect of intensity fading and achieve smooth transitions between consequent sections. After intensity correction, a series of cross-sectional images were converted into images in coronal plane and filtered with 3D bilateral filter in order to remove noise while preserving internal tissue structures. After this step, certain number of coronal plane images were extracted and loaded to the 3D Aligner software, in which they are to be aligned and merged. This task was solved by combination of scale invariant feature transform method, normalized cross-correlation (NCC) and shape-based alignment. For alignment of sections, a combination of median and threshold filters should be applied to remove remained noise. For whole mount 3D reconstruction of brain and kidney imaging follows the same steps as mentioned above. At last, the SOCM data aligned as an entire volume was rendered using 3D visualization software.

### Image processing for quantitative analysis of kidney inner structure

In order to analyze inner structure kidney including vessel network, it was required the additional image processing as shown in Fig. S8 of the Supplementary Materials. All aligned kidney data along the z-direction were initially passed through Support Vector Machine (SVM) algorithm. Based on manually selected positive samples and automatically selected negative samples from same images, classifier was trained and applied to remained images. The software named as vessel segmentation set the thresholding values to exclusively obtain the vessel information. Small discontinuities in blood vessel network were fixed using curve fitting. As a result, 3D structure showing probability of blood vessel to be presented in every voxel was obtained. Hole segmentation in renal structure started with automatic selection of initial image according to the following criteria: the manual search finds out biggest area of hole, segmented by Otsu’s method. This hole was then traced through the whole image stack. Obtained structure was smoothed with interpolation. Finally, data was processed to remove small objects. Processed date set was analyzed to get quantitative parameters, such as volume of hole, radius of blood vessels, and volume of whole kidney.

The distribution of vessel was analyzed for normal kidney and UUO models. First, the sum projection image along the axial direction was obtained after the images were rescaled. The intensity values were then radially summed for every 10 degrees, and corresponding result was plotted in polar coordinate while displaying the change in the distribution of blood vessels between normal and UUO models with respect to angle. In addition, angle distribution of blood vessel around main artery indicated with red dot was analyzed in the same manner. Here, the relative intensity value from 0 to 1 was used by dividing all intensity values by maximum intensity value of the normal kidney.

## Supplementary Information

Supplementary Information.

Supplementary Video1.

Supplementary Video2.

Supplementary Video3.
