# Supplementary material for: Serial optical coherence microscopy for label-free volumetric histopathology
Source: Sci Rep. 2020 Apr 21;10:6711. doi: 10.1038/s41598-020-63460-3 (PMC7174280; doi:10.1038/s41598-020-63460-3)
Supplement: Supplementary file 1 — Supplementary Information. [file 41598_2020_63460_MOESM1_ESM.docx]

**Supplementary Information for:**

**Serial optical coherence microscopy for label-free volumetric histopathology**

Eunjung Min^1,†^, Sungbea Ban^2,†^, Junwon Lee^2^, Andrey Vavilin^2^, Songyee Baek^2^, Sunwoo Jung^2^, Yujin Ahn^2^, Kibeom Park^2^, Sungwon Shin^2^, Sohyun Han^3^, Hyungjoon Cho^2^, Whaseon Lee-Kwon^2^, Jeehyun Kim^4^, C. Justin Lee^5^, and Woonggyu Jung^2,*^

^1^Max Planck Institute for Biological Cybernetics, 72076 Tübingen, Germany

^2^Department of Biomedical Engineering, Ulsan National Institute of Science and Technology (UNIST), Ulsan 44919, Republic of Korea

^3^Martinos Center for Biomedical Imaging, Charlestown, MA 02129, United States

^4^School of Electronics, Kyungpook National University, Daegu 41566, Republic of Korea

^5^Center for Cognition and Sociality, Institute for Basic Science, Daejeon 34126, Republic of Korea

^†^ These authors contributed equally to this work

* wgjung@unist.ac.kr

**Supplementary Information Contents:**

**Figures**

- **Supplementary Figure S1:** Volumetric datasets of brain slices obtained by SOCM in coronal plane
- **Supplementary Figure S2:** Flow diagram of 3D reconstruction algorithm for quantitative whole brain imaging
- **Supplementary Figure S3:** Anatomical comparison of brain tissue using various imaging modalities
- **Supplementary Figure S4:**Comparison of anatomical structures at axial and sagittal brain sections using various imaging modalities
- **Supplementary Figure S5:** High-resolution SOCM images of bundle of myelin fibers
- **Supplementary Figure S6:** Comparison of normal kidney morphology between stained histology and 3D reconstructed SOCM image
- **Supplementary Figure S7:** Volumetric datasets of kidney slices obtained by SOCM in transverse plane
- **Supplementary Figure S8:** Flow diagram for 3D reconstruction and analysis of kidney vessels
- **Supplementary Figure S9:** Vessel network destruction in UUO model

**Videos**

- **Supplementary Video S1:** Image reconstruction process of whole brain using SOCM
- **Supplementary Video S2:** Thee-dimensional visualization of whole brain
- **Supplementary Video S3:** Thee-dimensional visualization of whole kidney with vessel network

**
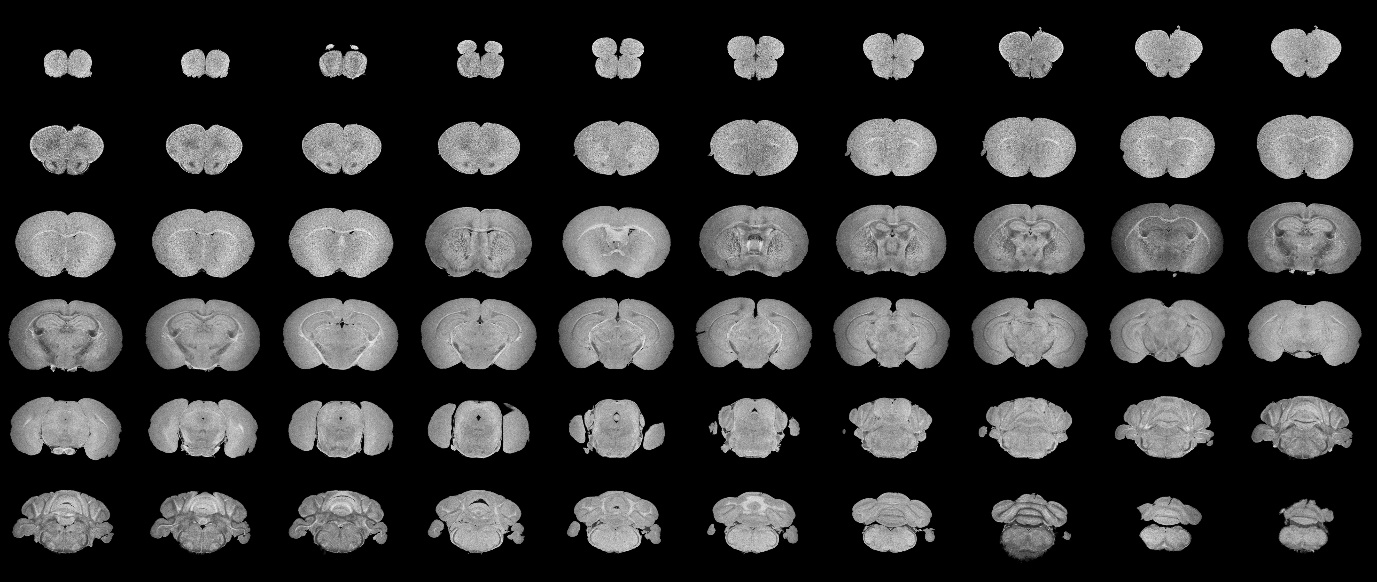
**

Supplementary Figure S1. Volumetric datasets of brain slices obtained by SOCM in coronal plane. The brain was serially imaged as 60 coronal volumetric sections ranging from olfactory to cerebellum. The axial and lateral resolution in air were 9.9 μm and 10.7 μm, respectively, and confocal parameter was 0.14 mm. The sampling resolution in xy and z-directions are 5 μm, 3.3 μm, respectively. The thickness of a single brain slice was 200 μm.


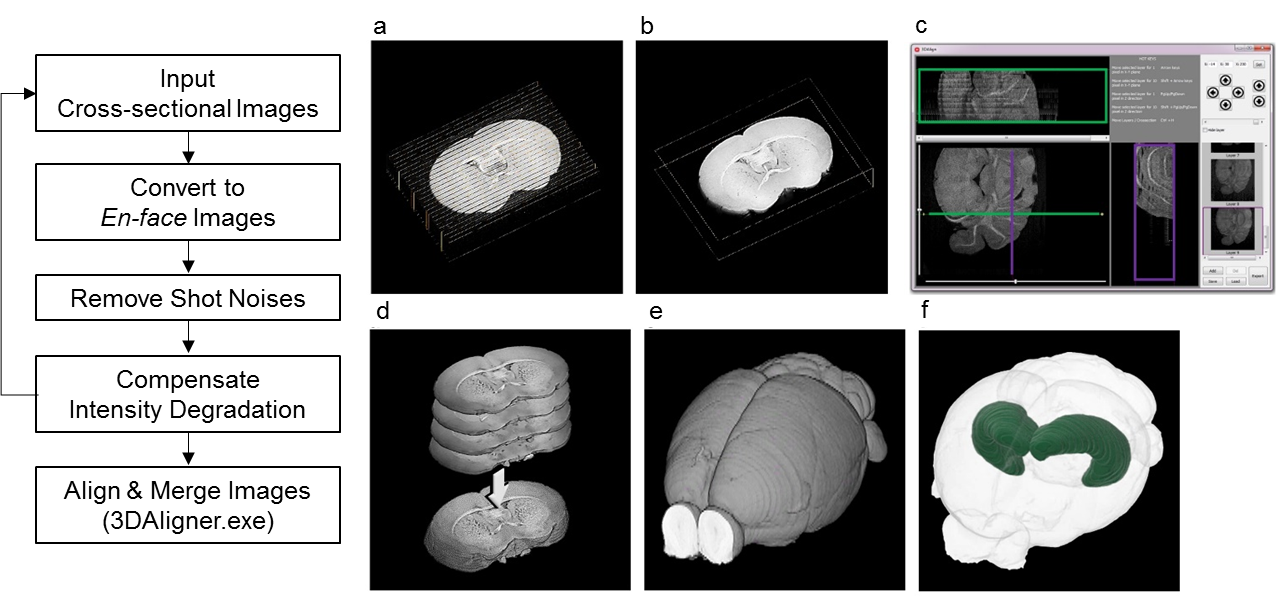


Supplementary Figure S2. Flow diagram of 3D reconstruction algorithm for quantitative whole brain imaging. SOCM dataset is processed as the flow diagram shows. a, Firstly, cross sectional images are transformed into b, en-face images. c, The images are then loaded on a lab-built software, named 3DAligner.exe, which is used to align and merge the series of 3D SOCM datasets in 3D. d, The concept of 3D aligning and merging is illustrated. e, After aforementioned process is completed, an entirely reconstructed optical whole brain image dataset is obtained. f, The dataset can be used to measure brain regional volume for a specific application.


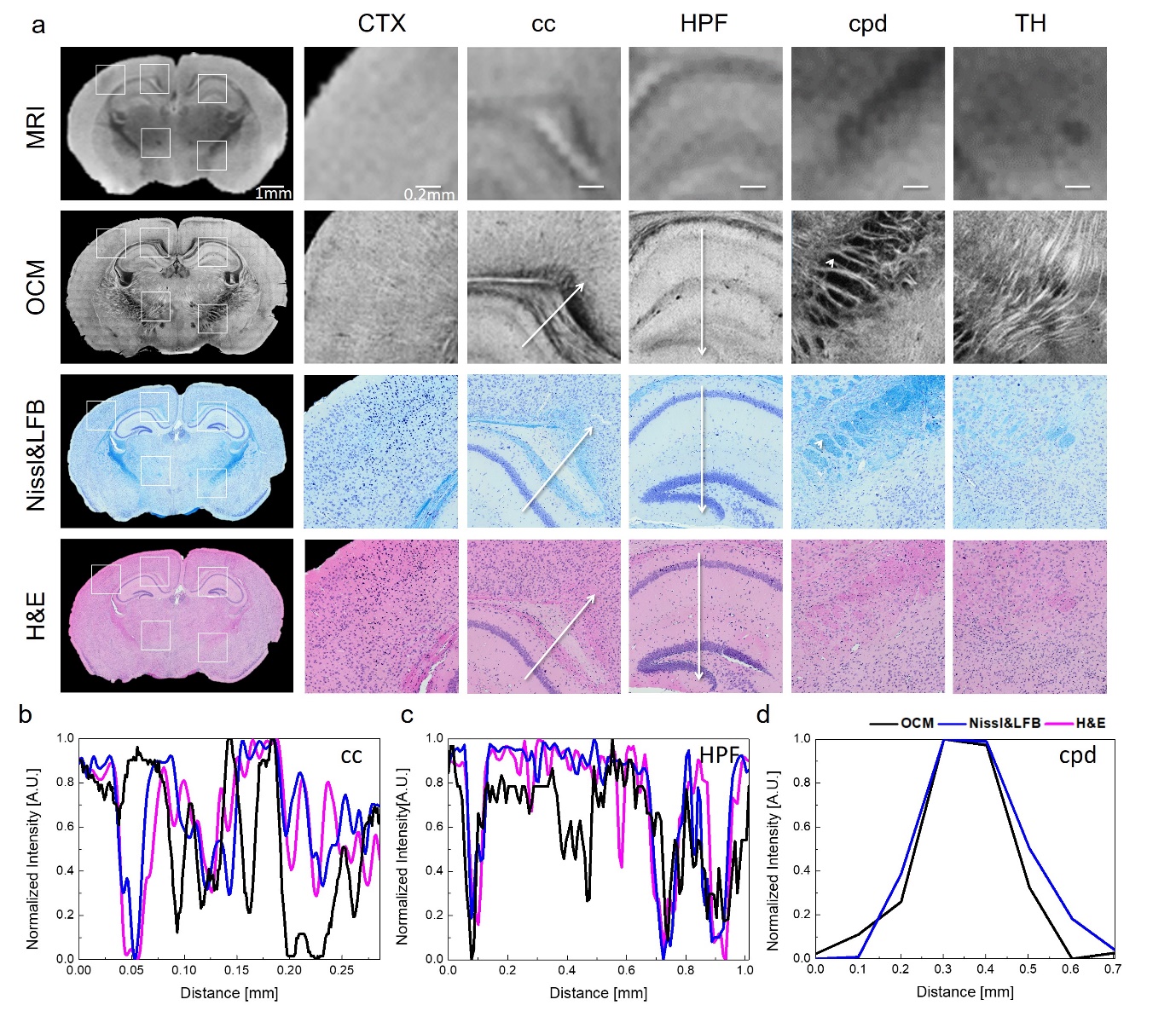


Supplementary Figure S3: Anatomical comparison of brain tissue using various imaging modalities. a. Anatomical structure of coronal sections of brain were compared using 7T MRI, OCM (0.5NA), and histological sections stained by hematoxylin and eosin (H&E), Nissl and Luxol fast blue. For accurate comparison, same brain was used. CTX, cortex; cc, corpus callosum; HPF, hippocampus formation; cpd, cerebral peduncle; TH, thalamus. b-d. The line profile for the region indicated by white arrows.

**
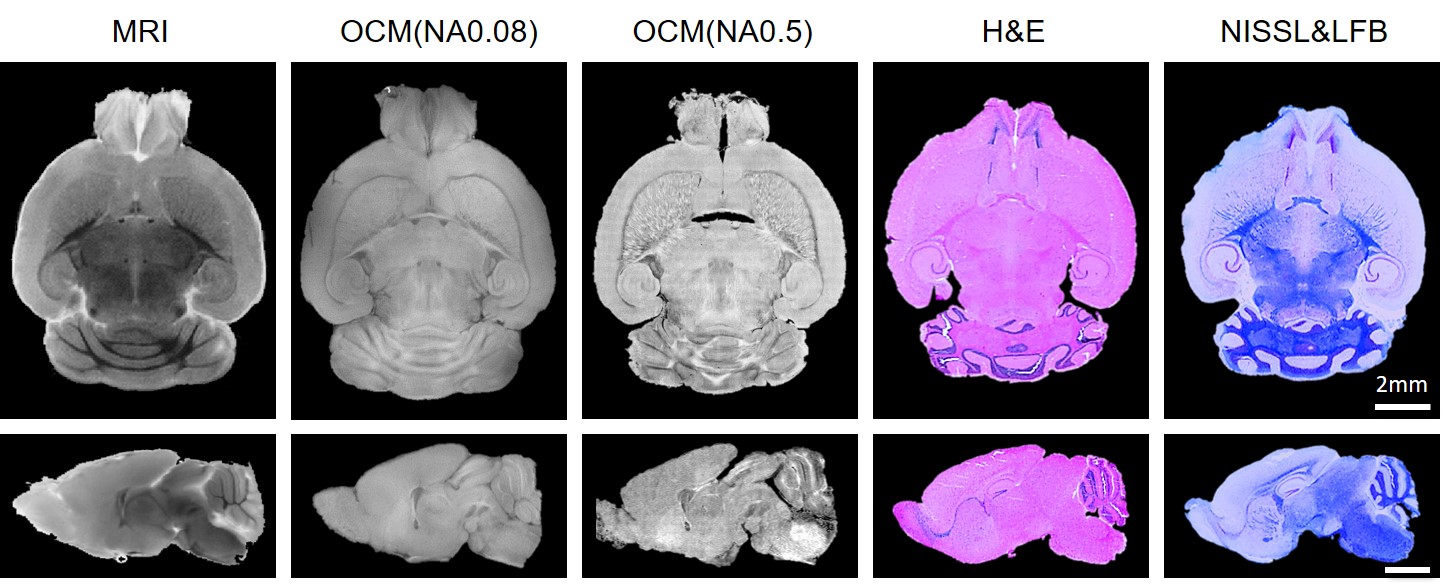
**

Supplementary Figure S4. Comparison of anatomical structures at axial and sagittal brain sections using various imaging modalities. Anatomical structure of axial and sagittal sections of brain were compared using 7T MRI, OCM, and histological sections stained by hematoxylin and eosin (H&E), Nissl and Luxol fast blue. For accurate comparison, same brain was used.

**
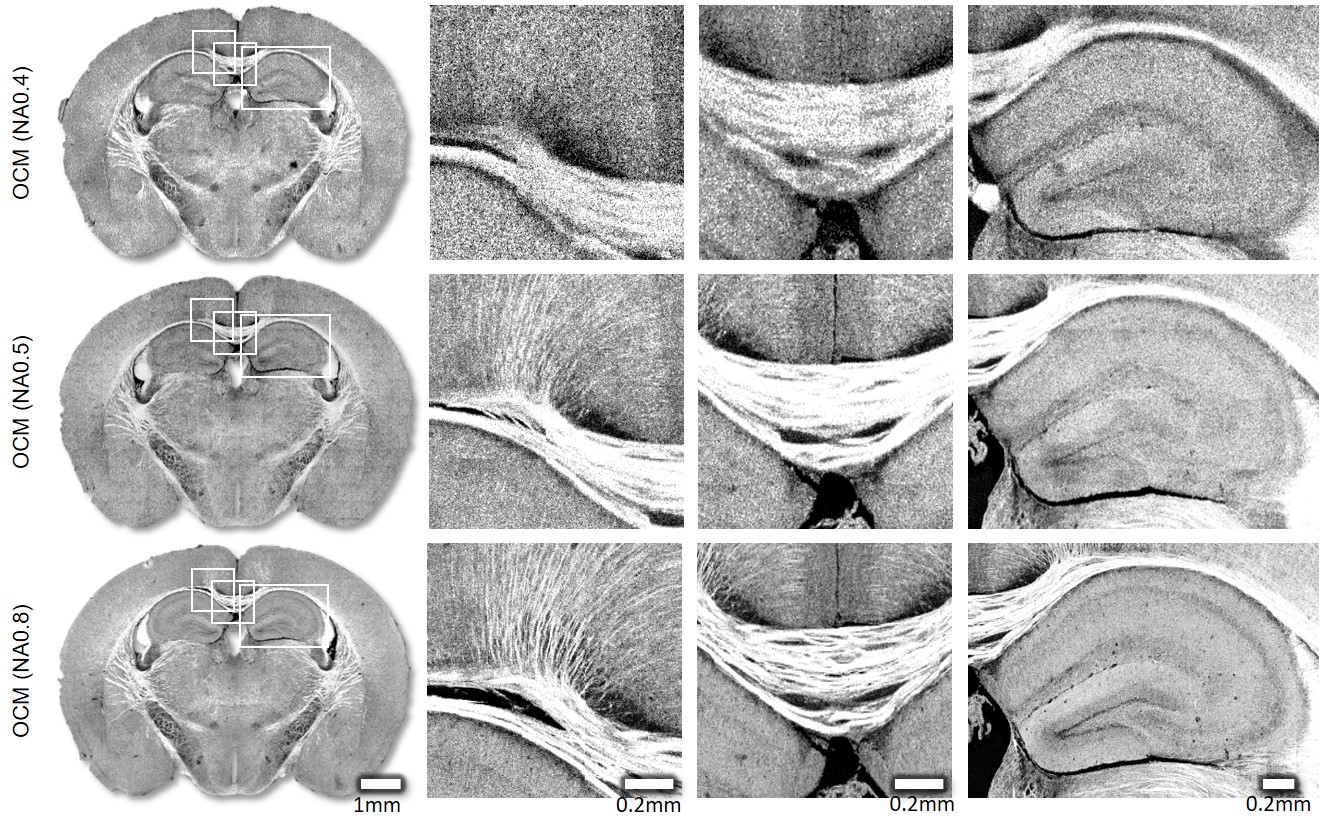
**

Supplementary Figure S5: High-resolution SOCM images of bundle of myelin fibers. The myelin fibers in the fiber tracts are imaged by using various NA objective lenses.

**
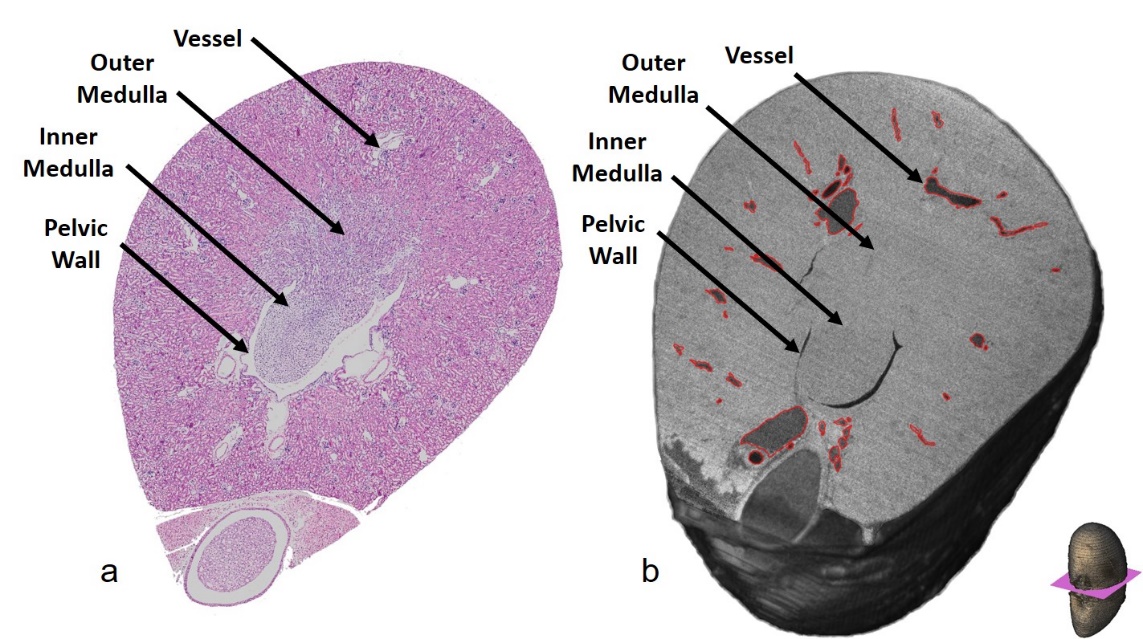
**

Supplementary Figure S6. Comparison of normal kidney morphology between stained histology and 3D reconstructed SOCM image. a, histology image of kidney stained by hematoxylin and eosin (H&E). b, Reconstructed SOCM kidney morphology in transverse direction denoted by purple indicating plane.


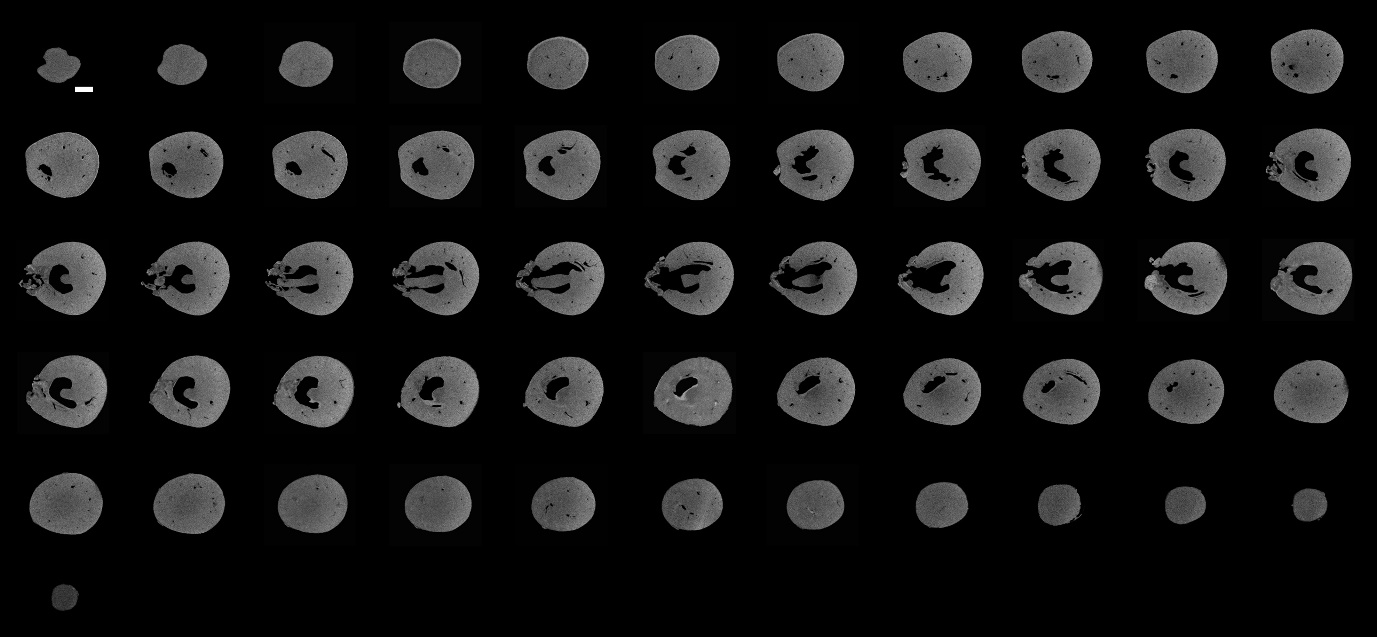

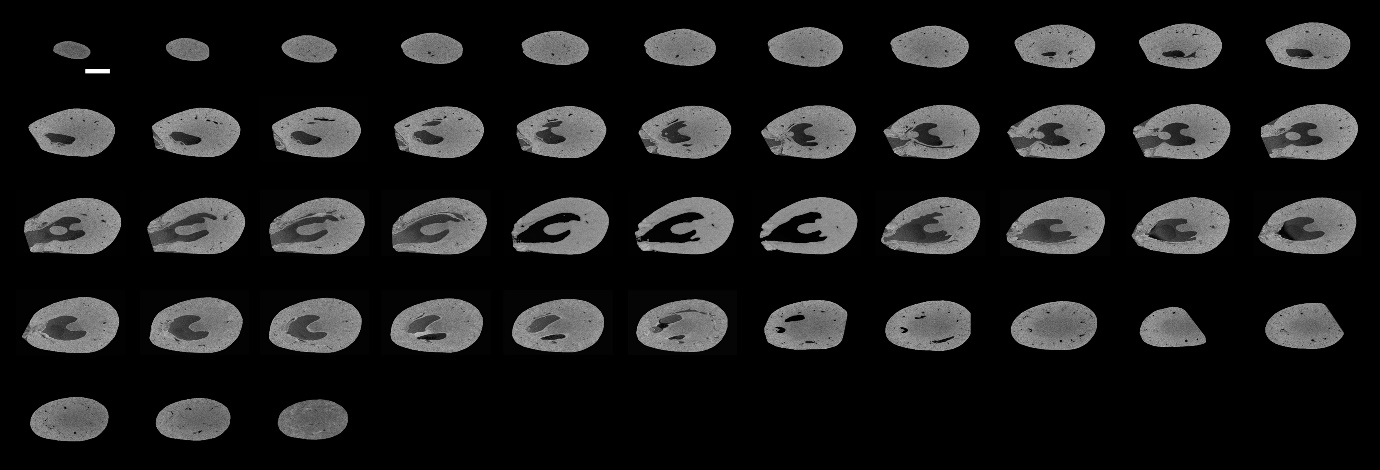

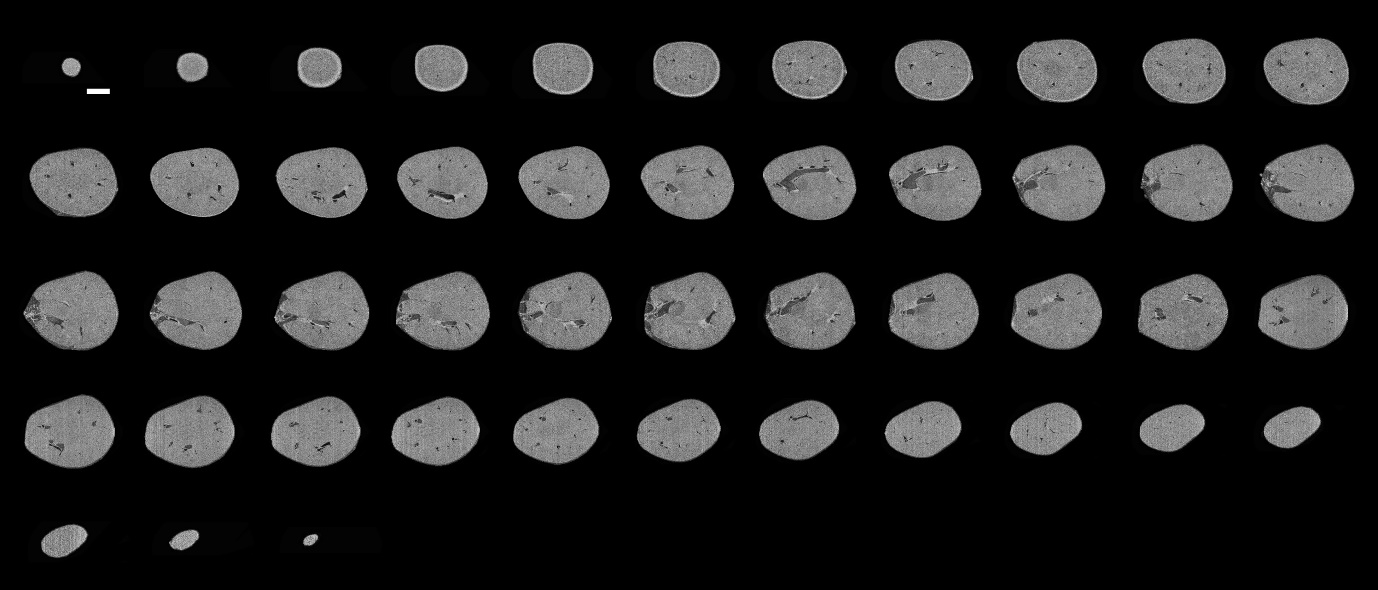


Supplementary Figure S7. Volumetric datasets of kidney slices obtained by SOCM in transverse plane. The kidney was serially imaged as 47 coronal volumetric sections in wild (Top), 56 sections for 3 day (Middle), and 47 sections for 5 day of UUO (Bottom). Scale bar is 1.5 mm. The thickness of a single kidney slice was 200 μm and 125 μm for normal and UUO model, respectively.


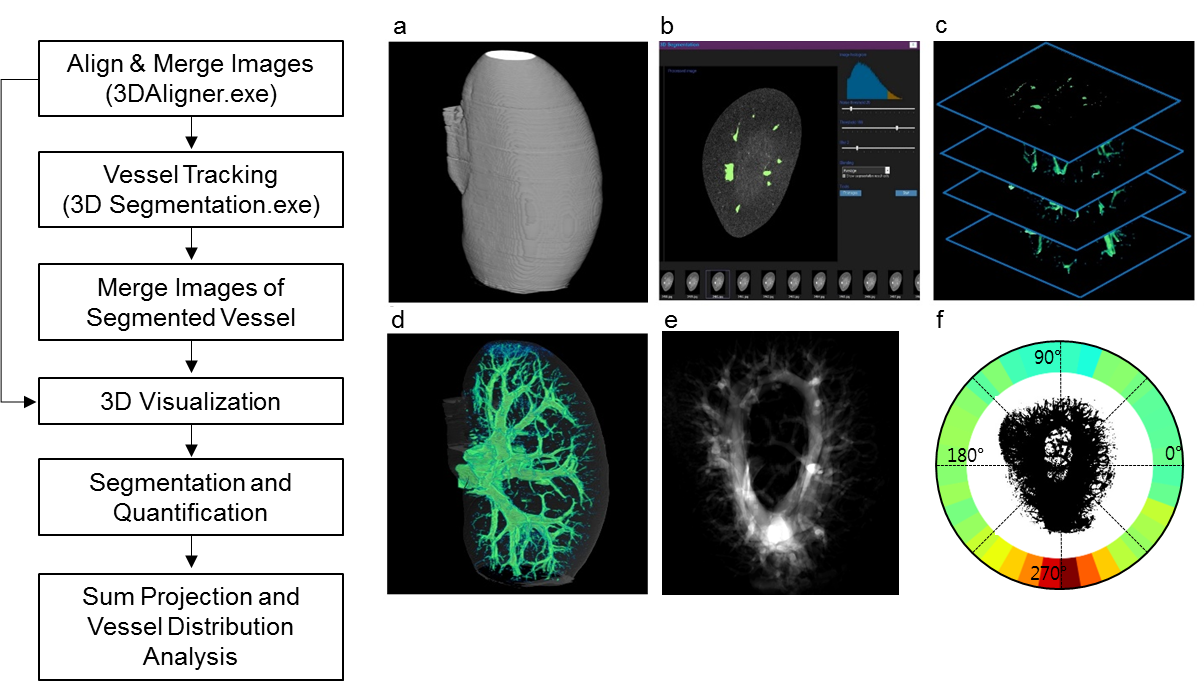


Supplementary Figure S8. Flow diagram for 3D reconstruction and analysis of kidney vessels. SOCM dataset is processed as the flow diagram shows. a, The whole image of kidney is reconstructed using 3DAligner.exe. b, The images are then loaded on a lab-built software, named 3D Segmentation.exe, which is used to align and merge segmented vessel images in 3D. c, The concept of 3D aligning and merging is illustrated. d, After aforementioned process is completed, a blood vessel image of whole kidney is reconstructed. e, The sum projection method was applied to entire 3D vessel image to analyze vessel distribution in 3D. f, The angle distribution of blood vessel was investigated in polar coordinate.


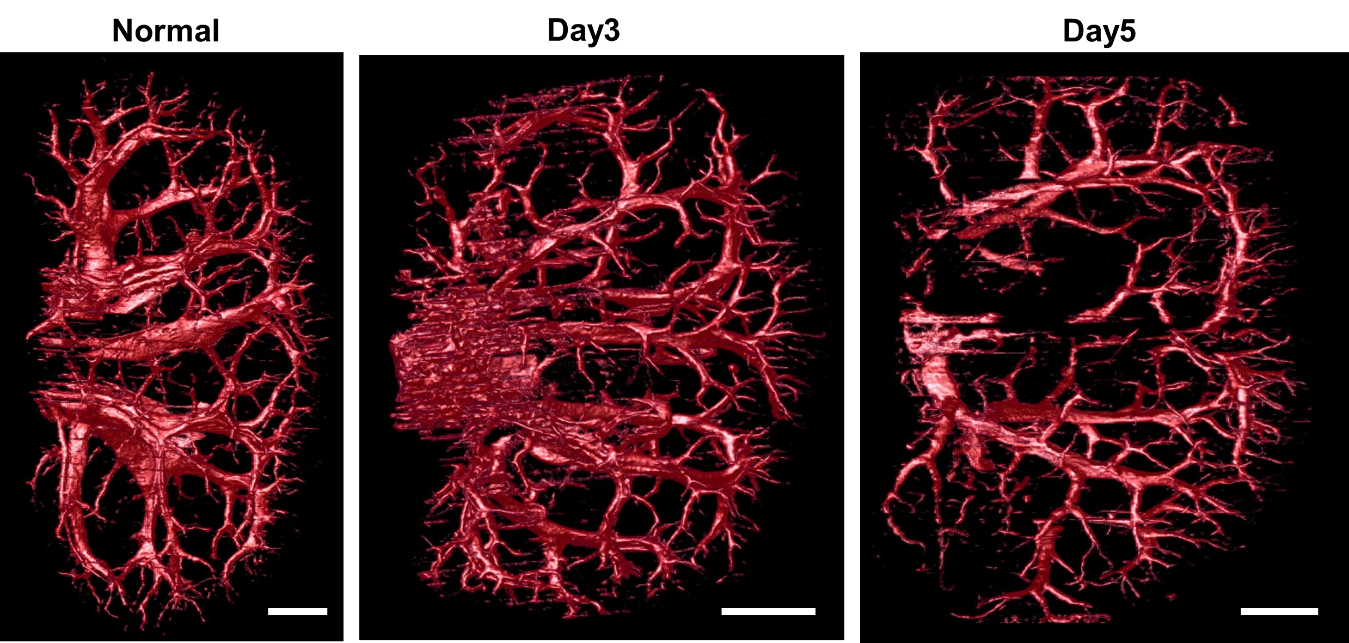


Supplementary Figure S9. Vessel network destruction in UUO model. Visualization of blood vessel architecture in whole kidney. Gradually constricted vessels are main indicator that deteriorate renal function. Damaged renal vessels are clearly presented in times series of UUO development. Scale bar is 1 mm.
